# The Cysteine Protease Legumain Is Upregulated by Vitamin D and Is a Regulator of Vitamin D Metabolism in Mice

**DOI:** 10.3390/cells13010036

**Published:** 2023-12-22

**Authors:** Karl Martin Forbord, Meshail Okla, Ngoc Nguyen Lunde, Tatjana Bosnjak-Olsen, Guro Arnekleiv, Daniel Hesselson, Harald Thidemann Johansen, Jonathan C. Y. Tang, Moustapha Kassem, Rigmor Solberg, Abbas Jafari

**Affiliations:** 1 Section for Pharmacology and Pharmaceutical Biosciences, Department of Pharmacy, University of Oslo, P.O. Box 1068 Blindern, 0316 Oslo, Norway; k.m.f.forbord@farmasi.uio.no (K.M.F.);; 2 Department of Endocrinology and Metabolism, Odense University Hospital, University of Southern Denmark, 5230 Odense, Denmark; 3 Department of Community Health Sciences, College of Applied Medical Sciences, King Saud University, Riyadh 11433, Saudi Arabia; 4 Centenary Institute, Faculty of Medicine and Health, The University of Sydney, Sydney, NSW 2006, Australia; 5 Bioanalytical Facility, Norwich Medical School, University of East Anglia, Norwich NR4 7TJ, UK; jonathan.tang@uea.ac.uk; 6 Clinical Biochemistry, Norfolk and Norwich University Hospital, Norwich NR4 7UY, UK; 7 Department of Cellular and Molecular Medicine, University of Copenhagen, Blegdamsvej 3B, 2200 Copenhagen, Denmark

**Keywords:** asparaginyl endopeptidase, legumain, metabolism, proteolysis, vitamin D

## Abstract

Legumain is a lysosomal cysteine protease that has been implicated in an increasing amount of physiological and pathophysiological processes. However, the upstream mechanisms regulating the expression and function of legumain are not well understood. Here, we provide in vitro and in vivo data showing that vitamin D_3_ (VD_3_) enhances legumain expression and function. In turn, legumain alters VD_3_ bioavailability, possibly through proteolytic cleavage of vitamin D binding protein (VDBP). Active VD_3_ (1,25(OH)_2_D_3_) increased legumain expression, activity, and secretion in osteogenic cultures of human bone marrow stromal cells. Upregulation of legumain was also observed in vivo, evidenced by increased legumain mRNA in the liver and spleen, as well as increased legumain activity in kidneys from wild-type mice treated with 25(OH)D_3_ (50 µg/kg, subcutaneously) for 8 days compared to a control. In addition, the serum level of legumain was also increased. We further showed that active legumain cleaved purified VDBP (55 kDa) in vitro, forming a 45 kDa fragment. In vivo, no VDBP cleavage was found in kidneys or liver from legumain-deficient mice (*Lgmn*^−/−^), whereas VDBP was cleaved in wild-type control mice (*Lgmn*^+/+^). Finally, legumain deficiency resulted in increased plasma levels of 25(OH)D_3_ and total VD_3_ and altered expression of key renal enzymes involved in VD_3_ metabolism (CYP24A1 and CYP27B1). In conclusion, a regulatory interplay between VD_3_ and legumain is suggested.

## 1. Introduction

Legumain is a cysteine endopeptidase with strict specificity for hydrolysis of peptide bonds C-terminally of asparagine residues [1], hence the synonym asparaginyl endopeptidase (AEP). Although mainly lysosomal, legumain has also been shown to be secreted and detectable in plasma. In addition, legumain is postulated to have autocrine/paracrine functions (reviewed in [2]). Legumain is highly expressed in the kidneys, liver, and spleen [3] and is described as being involved in the pathogenesis of several disorders, such as cardiovascular diseases (reviewed in [2]). We have previously shown that legumain expression is altered in the bone microenvironment of patients with osteoporosis [4]. An increasing number of proteins have been identified as substrates of legumain (reviewed in [2]). However, despite the increased knowledge of legumain substrates, mechanisms of action, and roles in the pathogenesis of different malignant and non-malignant diseases, the upstream mechanisms regulating legumain expression are not well understood.

Vitamin D_3_ (VD_3_) is a hormone involved in different biological processes such as calcium homeostasis, immune regulation, as well as cell growth and differentiation [5]. VD_3_ is primarily synthesized in the skin upon exposure to sunlight but can also be obtained from dietary sources. VD_3_ is then transported to the liver, where it undergoes hydroxylation by the vitamin D 25-hydroxylase (CYP2R1), resulting in the formation of 25-hydroxyvitamin D_3_ (25(OH)D_3_; calcidiol), which is the most abundant circulating form of VD_3_. In the kidneys, 25(OH)D_3_ undergoes further hydroxylation by 1α-hydroxylase (CYP27B1), generating the active form of VD_3_, i.e., 1α,25-dihydroxyvitamin D_3_ (1,25(OH)_2_D_3_; calcitriol) [6,7]. In addition, both 25(OH)D_3_ and 1,25(OH)_2_D_3_ can be catabolized through 24-hydroxylation by CYP24A1 to form the inactive 24,25(OH)_2_D_3_ and 1,24,25(OH)_3_D_3_ metabolites, respectively [8]. Furthermore, extra-renal expression of both CYP27B1 [9,10,11] and CYP24A1 [12,13,14] have been demonstrated, indicating a paracrine role of VD_3_ metabolites in some tissues (reviewed in [15]). The active 1,25(OH)_2_D_3_ metabolite exerts its biological effects through interaction with the nuclear vitamin D receptor (VDR) [16], which, upon ligand binding, heterodimerizes with the retinoid X receptor and translocates to the nucleus to alter transcription of target genes through binding to specific DNA sequences known as vitamin D-responsive elements (VDRE) [17,18].

VD_3_ is a fat-soluble molecule and is hence transported in the bloodstream bound to carrier proteins, mainly vitamin D binding protein (VDBP). VDBP is synthesized in the liver and secreted to the circulation, where it binds and transports the majority of VD_3_ metabolites [19,20]. VDBP is partially filtered in the glomerulus and subsequently reabsorbed in the proximal tubuli through megalin/cubilin-mediated internalization [21]. After reabsorption, VDBP is proteolytically cleaved in the endolysosomal compartments, and VD_3_ is released for hydroxylation. Legumain has been shown to cleave VDBP in incubates with purified bovine legumain [22], although this has not been corroborated in vivo.

Both VD_3_ and legumain regulate bone homeostasis; thus, we aimed to investigate potential interactions between VD_3_ and legumain. Using in vitro and in vivo studies, we showed that VD_3_ is an upstream regulator of legumain expression and that the presence or absence of legumain alters the processing of VDBP, as well as metabolism and the circulating levels of VD_3_.

## 2. Materials and Methods

### 2.1. Chemicals and Reagents

Bovine serum albumin, CHAPS (3-((3-cholamidopropyl) dimethylammonium)-1-propanesulfonate), citric acid, DAPI, dexamethasone, 1,25(OH)_2_D_3_, 25(OH)D_3_ (for in vitro experiments), VDBP (Gc-globulin) from human plasma (Catalog # G8764), DTT (dithiothreitol), β-glycerophosphate, L-ascorbic acid, Na_2_HPO_4_, Na_2_EDTA, TRI Reagent^®^, Tween^®^ 20, and p-nitrophenyl phosphate were purchased from Sigma-Aldrich, St. Louis, MO, USA. Ethanol (96%—rectified) was purchased from Antibac, Asker, Norway. For in vivo treatment with 25(OH)D_3_, D3-Vicotrat^®^ (Hilden, Germany) was used. The Z-Ala-Ala-Asn-AMC peptide substrate was purchased from Bachem, Bubendorf, Switzerland. Chameleon^®^ Duo Protein Ladder, donkey anti-goat IR Dye 680LT and 800CW, donkey anti-mouse IR dye 800CW, donkey anti-rabbit IR dye 680LT and 800CW, and Odyssey^®^ Blocking Buffer were obtained from LI-COR, Cambridge, UK. High-Capacity cDNA Reverse Transcription Kit (Catalog # 4368814), Minimal Essential Media (MEM), and Power SYBR™ Green PCR Master Mix (Catalog # 4367659) were obtained from Thermo Fischer Scientific, Waltham, MA, USA. RNeasy^®^ Plus Mini Kit and Buffer RLT Plus were purchased from QIAGEN, Hilden, Germany. Trans-Blot^®^ Turbo™ Mini-size nitrocellulose membrane and Trans-Blot^®^ Turbo™ Transfer System were purchased from Bio-Rad, Copenhagen, Denmark. RNeasy^®^ Plus Mini Kit was purchased from QIAGEN, Hilden, Germany. GentleMACS™ M Tubes were purchased from Miltenyi Biotec, Bergisch Gladbach, Germany. EconoSpin^®^ spin columns were purchased from Epoch Life Science, Missouri City, TX, USA. Primers for mouse legumain, VDBP, CYP27B1, CYP24A1, RPLP0, GAPDH, and β-actin were purchased from Ebersberg, Germany. Bovine mature active legumain (36 kDa) was acquired as previously described [23].

### 2.2. Identification of Putative Vitamin D-Responsive Elements in the LGMN Promoter Region

The nucleotide sequence cut-off of the human legumain (LGMN) gene promoter (accession no.: NM_005606) was set to 1485 base pairs downstream and 15 base pairs upstream of the transcription start site. The nucleotide sequence was retrieved using the Sequence Retrieval Tool in the Eukaryotic Promoter Database (https://epd.epfl.ch (accessed on 20 September 2022)). Putative vitamin D-responsive elements (VDRE) were identified using the PROMO database [24,25] with the maximum matrix dissimilarity rate set to 15.

### 2.3. Cell Culturing

For cell culture experiments, human bone marrow-derived mesenchymal stromal cells stably overexpressing the catalytic subunit of human telomerase (hBMSC-TERT cell line, RRID:CVCL_Z017; further referred to as hBMSC) were used [4]. The cells were grown in a basal medium containing Minimal Essential Media (MEM) with L-glutamine, 10% (*v*/*v*) foetal bovine serum, 1% penicillin (100 U/mL), and streptomycin (100 µg/mL). The cells were seeded at a density of 20,000 cells/cm^2^, and at 80% confluence, the cells were differentiated using osteoblastic induction medium containing basal medium supplemented with 10 mM β-glycerophosphate, 50 µg/mL L-ascorbic acid, 10 nM dexamethasone [4], and 1,25-dihydroxyvitamin D_3_ (0–100 nM) or 25(OH)D_3_ (0–1000 nM) in ethanol solution (for controls, an equivalent volume of ethanol was used) for seven days. The induction medium was renewed every three or four days. Monoclonal legumain over-expressing HEK293 (M38L) and HEK293 (human embryonic kidney 293; RRID:CVCL_0045) cells were cultured as previously described [26]. In brief, the cells were seeded at a density of 5 × 10^6^ cells/75 cm^2^ flask and cultured in Dulbecco′s Modified Eagle′s Medium with 10% (*v*/*v*) foetal bovine serum. G418 (800 μg/mL) was added to the culture medium of M38L cells.

### 2.4. Harvesting of Cell-conditioned Media and Lysates

Cell-conditioned media were collected and centrifuged at 800 rpm for 5 min at 4 °C, and the supernatants were frozen at −20 °C. Adherent cells were washed with PBS before harvesting in legumain lysis buffer (100 mM sodium citrate, 1 mM disodium-EDTA, 1% n-octyl-β-D-glucopyranoside, pH 5.8) for quantitation of legumain activity or Buffer RLT Plus for mRNA isolation. Cell lysates harvested in legumain lysis buffer were frozen (−70 °C) and thawed (30 °C) three times before centrifugation at 10,000× *g* for 5 min, and the supernatants were frozen at −20 °C or directly analyzed. Total protein concentrations in cell lysates were determined by measuring absorbance at 595 nm in a microplate reader (Wallac Victor^®^ 3™ or Wallac Victor^®^ Nivo™, Perkin Elmer, Boston, MA, USA) according to Bradford [27] and the manufacturer. Bovine serum albumin (0–400 µg/mL) was used to generate a standard curve for the calculation of total protein concentrations. All measurements were performed in triplicates.

### 2.5. Legumain-Deficient Mice

Legumain-deficient (*Lgmn*^−/−^) mice were produced using CRISPR/Cas9 gene targeting in C57BL/6J mouse embryos following established molecular and animal husbandry techniques [28]. A single guide RNA (sgRNA) was designed to target within exon 1 of *Lgmn* (target with protospacer-associated motif underlined GGATGGAGGCAAGCACTGGGTGG) and co-injected with polyadenylated Cas9 mRNA into C57BL/6J zygotes. Microinjected embryos were cultured overnight and introduced into pseudo-pregnant foster mothers. Pups were screened by PCR and Sanger sequencing of ear-punch DNA and a founder mouse was identified that carried a 10 bp frame-shift deletion in exon 1. The targeted allele was maintained by breeding on a C57BL/6J background.

### 2.6. Treatment of Mice with 25(OH)D_3_ and Tissue Harvesting

Twelve-week-old female legumain wild-type (*Lgmn*^+/+^) and legumain-deficient (*Lgmn*^−/−^) mice were bred and housed under standard conditions (21 °C, 55% relative humidity) on a 12 h light/dark cycle. The mice were injected subcutaneously (s.c.) on day 0, 2, 4, and 7 with 50 µg/kg 25(OH)D_3_ (Vicotrat^®^) in 5% DMSO and 95% saline (n = 7) or an equal volume of vehicle (5% DMSO and 95% saline control, n = 7)). After the final injection, the mice were fasted overnight and anesthetized before blood was collected by retro-orbital bleeding and plasma was obtained after centrifugation and frozen at −80 °C. Subsequently, the mice were euthanized, and kidneys, liver, and spleen were collected, snap-frozen in liquid nitrogen, and stored at −80 °C. Mice experiments were carried out in accordance with permissions issued by the Danish Animal Experiments Inspectorate (2022-15-0201-01225). Tissue samples were homogenized in gentleMACS™ M Tubes (Miltenyi Biotec) using a gentleMACS™ Octo Dissociator (Miltenyi Biotec) in either TRI Reagent^®^ (Sigma) or lysis buffer for subsequent mRNA isolation or protein analysis, respectively.

### 2.7. Legumain Activity Measurement

Cleavage of the peptide substrate Z-Ala-Ala-Asn-AMC was used to measure the proteolytic activity of legumain, as previously described [29]. In brief, 20 µL of cell lysates or tissue homogenates, 100 µL assay buffer (39.5 mM citric acid, 121 mM Na_2_HPO_4_, 1 mM Na_2_EDTA, pH 5.8, 1 mM DTT, and 0.1% CHAPS) and 50 µL peptide substrate solution (final concentration 10 µM) were added in black 96-well microtiter plates (Corning Life Science, Lowell, MA, USA). Kinetic measurements based on the increase in fluorescence (360EX/460EM) for 10 or 60 min were performed at 30 °C in a microplate reader (Wallac Victor^®^ 3™ or Wallac Victor^®^ Nivo™ (Perkin Elmer)).

### 2.8. Immunoblotting and Enzyme-Linked Immunosorbent Assay (ELISA)

Gel electrophoresis and immunoblotting were performed by loading 15–20 µg of total protein using NuPAGE 4–12% gels (Life Technologies, Carlsbad, CA, USA) and NuPAGE MOPS SDS running buffer prior to transfer to a nitrocellulose membrane (Trans-Blot^®^ Turbo™ Mini-size nitrocellulose) in the Trans-Blot^®^ Turbo™ Transfer System for 30 min. The membranes were blocked for 1 h at room temperature with Odyssey^®^ Blocking Buffer and probed with polyclonal goat anti-human legumain (1:200, R&D Systems, Minneapolis, MN, USA, Catalog # AF2199, RRID: AB_416565), polyclonal rabbit anti-human/mouse VDBP (1:500, Bio-Techne, Minneapolis, MN, USA, Catalog # NBP1-88027, RRID: AB_11023579), monoclonal mouse anti-human VDBP (1:500, R&D Systems, Catalog # MAB3778, RRID: AB_2232276), monoclonal mouse anti-human GAPDH antibody (1:10,000, Santa Cruz Biotechnology Inc., Dallas, TX, USA, Catalog # sc-47724, RRID: AB_627678), or monoclonal mouse anti-human GAPDH (1:10,000, R&D Systems, Catalog # MAB5718, RRID: AB_10892505) antibody in Tris-buffered saline containing 0.1% Tween 20 (T-TBS) overnight at 4 °C. Membranes were subsequently washed 3–4 times in T-TBS buffer and incubated with donkey anti-mouse 800CW (1:10,000), donkey anti-goat, or donkey anti-rabbit IR Dye 680LT (1:10,000) or 800CW (1:10,000) for 1 h at room temperature. After another washing procedure, membranes were briefly dried and analyzed using Odyssey-CLx Imaging System (LI-COR).

Total human or mouse legumain ELISA kit was used to determine concentrations of legumain in cell-conditioned media or mice plasma, respectively, according to the manufacturer’s protocol (R&D Systems, Catalog # DY4769, RRID: AB_294369 and MyBioSource, Catalog # MBS9718081, RRID: AB_2943631, respectively). Plasma VDBP concentrations were measured using a mouse VDBP ELISA kit (R&D Systems, Catalog # DY4188-05, RRID: AB_2943630).

### 2.9. Quantitative PCR

Total RNA was extracted from cell lysates harvested in Buffer RLT Pluss using an RNeasy^®^ Plus Kit according to the manufacturer’s protocol or from tissue homogenates by chloroform phase separation and subsequent EconoSpin column purification (Epoch Life Science). RNA was quantified using Nanodrop™ (Thermo Scientific, Waltham, MA, USA) and stored at −80 °C until analysis. Complementary DNA (cDNA) was synthesized from 2 µg mRNA using the High-Capacity cDNA Reverse Transcription Kit (Thermo Fisher Scientific) and ProFlex™ 3 × 32-well thermal cycler (Applied Biosystems, Thermo Fisher Scientific, Waltham, MA, USA) and stored at −20 °C until analysis. Primers (Supplementary Table S1) were designed by the Primer Express software version 1 (Applied Biosystems, Thermo Fisher Scientific). Gene expressions were examined by real-time quantitative PCR (qPCR) using Power SYBR™ Green PCR Master Mix and the Applied Biosystems StepOnePlus™ Instrument with the accompanying software StepOne™ Version 2.3 (Applied Biosystems, Thermo Fisher Scientific). Gene expression was normalised against the geometric means of the CT values of housekeeping controls (RPLP0, GAPDH, β-actin, 18s) [30].

### 2.10. Measurement of Total VD_3_ Metabolites in Mouse Plasma

Vitamin D metabolite concentrations in mouse plasma were analyzed by liquid chromatography–tandem mass spectrometry (LC-MS/MS), as previously described [31]. The assays were calibrated using the National Institute of Science and Technology (NIST) standard reference material SRM972a. Total 25(OH)D_3_ was calculated from the sum of the measurements of VD_3_ and VD_2_ forms. Inter-assay coefficient of variation (CV) was <10.0% across the assay working range of 0.1 to 200.0 nmol/L.

1,25(OH)_2_D_3_ and 1,25(OH)_2_D_2_ were analyzed using a Waters Acquity Xevo TQXS LC-MS/MS system (Waters, Wilmslow, UK) [32]. Prior to analysis, plasma samples underwent immunoaffinity pretreatment to enrich the sample load, followed by derivatisation with Cookson-type dienophilic agents DAP-TAD (4-4-dimethylaminophenyl-1,2,4-triazoline-3,5-dione). The assays were calibrated using certified pure internal standards (Cerilliant, LGC). Inter-assay coefficient of variation (CV) was < 9.8% across the assay working range of 20 to 800.0 pmol/L.

All vitamin D metabolite assays met the requirements specified by vitamin D external quality assessment (DEQAS) scheme (http://www.deqas.org/; accessed on 30 January 2023). The 25OHD_3_ and 25OHD_2_ assays showed <6% accuracy bias against the Center for Disease Control and Prevention (CDC) reference measurement (RMP) target values on the DEQAS scheme.

### 2.11. Statistical Analysis

The data are represented as mean ± SEM. Student *t*-test, Kruskal–Wallis, Mann–Whitney, simple linear regression, and one-way or two-way ANOVA were performed when appropriate. Statistical significance was considered at *p* ˂ 0.05. All calculations were performed with GraphPad Prism (Version 9.0; GraphPad Software, Inc., San Diego, CA, USA).

## 3. Results

### 3.1. 1,25(OH)_2_D_3_ Regulates Legumain Expression in Pre-Osteoblastic Cells

**Figure 1:**
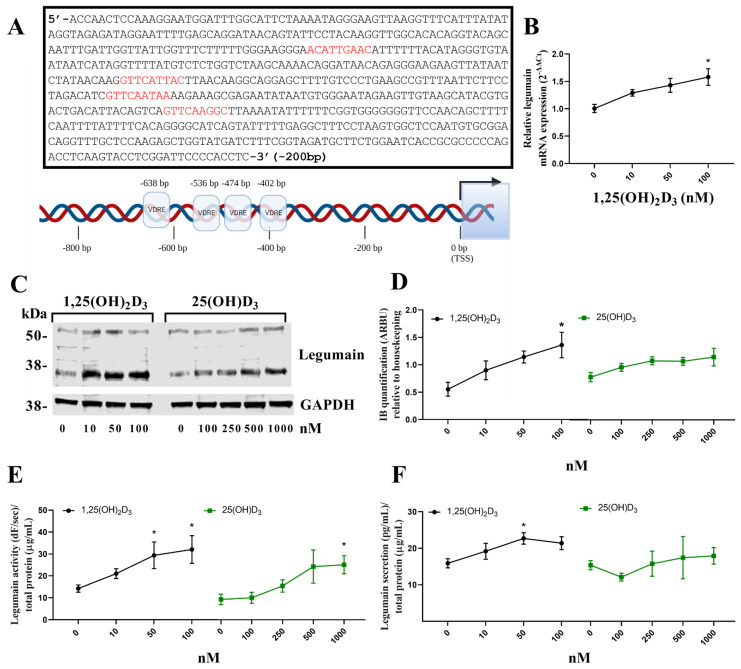
**Vitamin D3 increases legumain expression, activity, and secretion in pre-osteoblastic cells.** (**A**) The nucleotide sequence of the LGMN gene promoter region with annotations of potential vitamin D-responsive elements (VDRE; red) relative to the transcription start site (TSS). (**B**–**F**) Human BMSC-TERT cells (20,000 cells/cm^2^) were incubated with 1,25(OH)_2_D_3_ (**B**–**F**; 10, 50 or 100 nM), 25(OH)D_3_ (**C**–**F**; 100, 250, 500 or 1000 nM) or an equal volume of ethanol (control, 0 nM) in osteoblast induction medium for seven days before harvesting. (**B**) Legumain mRNA expression relative to housekeeping control (GAPDH) (2^−ΔΔCT^; n = 3). (**C**) One representative immunoblot of legumain (proform 56 kDa, mature form 36 kDa) and GAPDH (housekeeping) in cell lysates (n = 3). (**D**) Quantification of the 36 kDa mature legumain immunoband (IB) intensity as arbitrary units (ARBU) relative to GAPDH in immunoblots represented in C (n = 3). (**E**) Legumain activity (dF/s) in cell lysates adjusted for the total protein concentration (µg/mL) (n = 6–9). (**F**) Secreted legumain (pg/mL) in conditioned media measured by ELISA and adjusted for the total protein concentration in the corresponding cell lysates (n = 3–5). (**B**,**D**–**F**) Data represent mean ± SEM. (**B**,**D**) Kruskal–Wallis test. (**E**,**F**) One-way ANOVA. * *p* < 0.05 vs. 0 nM 1,25(OH)_2_D_3_ or 25(OH)D_3_. Numbers (n) represent individual biological replicates.

Given the role of VD_3_ in regulating the expression of several bone-related factors, we first aimed to investigate if VD_3_ could regulate the expression of the legumain encoding gene (LGMN). Analysis of the human LGMN gene promoter region using in silico analysis by the PROMO database revealed the presence of four potential vitamin D-responsive elements (VDRE) at the following nucleotide positions relative to the transcription start site: nucleotide −638 (dissimilarity (ds) = 4.62%), −536 (ds = 8.08%), −474 (ds = 8.93%), and −402 (ds = 6.93%) (Figure 1A). This suggested a possible regulation of LGMN expression by VD_3_. To test this hypothesis in a cell-based model, the effect of VD_3_ on legumain mRNA expression was investigated in osteogenic hBMSC cultures in the presence or absence of 1,25(OH)_2_D_3_ (10, 50 or 100 nM). We found a dose-dependent increase in legumain mRNA expression, reaching significance at 100 nM 1,25(OH)_2_D_3_ (Figure 1B).

To further investigate the effect of VD_3_ on legumain expression and proteolytic activity, osteogenic hBMSC were cultured with or without 1,25(OH)_2_D_3_ (10, 50, or 100 nM) or 25(OH)D_3_ (100, 250, 500, or 1000 nM) for 7 days. Immunoblot analysis showed a dose-dependent tendency of increased levels of 36 kDa mature legumain in the presence of 1,25(OH)_2_D_3_, reaching significance at 100 nM 1,25(OH)_2_D_3_ (Figure 1C,D). However, the expression was not significantly affected by 25(OH)D_3_. The effect of VD_3_ on legumain function was investigated by quantifying the proteolytic activity of legumain in the lysates. Increased legumain activity was observed in cells treated with 50 or 100 nM 1,25(OH)_2_D_3_ and with 1000 nM 25(OH)D_3_ (Figure 1E). As legumain can also be secreted and mediate autocrine/paracrine functions, we investigated whether VD_3_ could alter legumain secretion. ELISA measurements of legumain in the conditioned media showed increased legumain secretion by pre-osteoblastic cells in the presence of 50 nM 1,25(OH)_2_D_3_ (Figure 1F).

### 3.2. 25(OH)D_3_ Administration Increases Legumain Expression and Activity In Vivo

**Figure 2:**
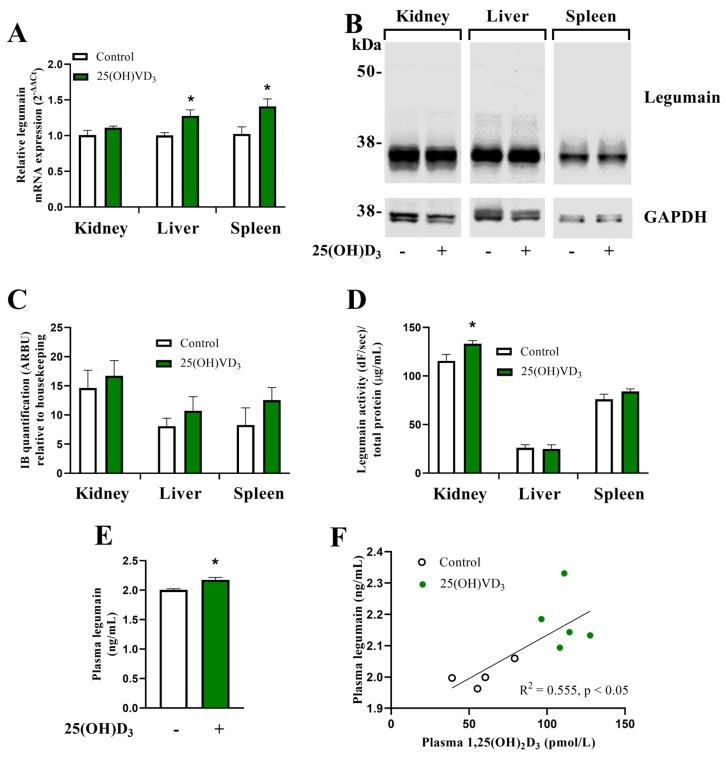
**Treatment with 25(OH)D_3_ increases legumain levels and activity in wild-type mice.** Wild-type mice (*Lgmn*^+/+^) were treated with 50 µg/kg 25(OH)D_3_ (n = 7) or an equal volume vehicle (n = 7, control) subcutaneously every two to three days (four times in total). Tissues were harvested 24 h after the final injection (day 8). (**A**) Legumain mRNA expression relative to the geometric mean of CT values of four housekeeping controls in kidney, liver, and spleen (2^−ΔΔCT^; n = 5). (**B**) One representative immunoblot of legumain and GAPDH in kidney, liver, and spleen (n = 3). (**C**) Quantification of the 36 kDa mature legumain immunoband (IB) intensity as arbitrary units (ARBU) relative to GAPDH (housekeeping) in kidney, liver, and spleen from immunoblots represented in (**C**) (n = 3). (**D**) Legumain activity (dF/s) in kidney, liver, and spleen adjusted for total protein concentration (μg/mL, n = 5). (**E**) Legumain plasma concentration (ng/mL) measured by ELISA (n = 5). (**F**) Correlation between legumain (ng/mL and 1,25(OH)_2_D_3_ (pmol/L) concentrations in plasma (n = 5). (**A**,**C**,**E**) Two-tailed unpaired Student’s *t*-test. (**D**) Mann–Whitney test. Data represent mean ± SEM. * *p* < 0.05. (**F**) Simple linear regression. Numbers (n) represent individual biological replicates.

To investigate whether VD_3_ also regulated the levels of legumain in vivo and whether legumain expression is important for vitamin D metabolism through VDBP processing (see below), high dose 25(OH)D_3_ (50 μg/kg) or vehicle was subcutaneously (sc) administrated to wild-type (*Lgmn*^+/+^) and legumain-deficient (*Lgmn*^−/−^) C57BL6/J mice for 8 days. Legumain deficiency in the kidneys, liver and spleen of *Lgmn*^−/−^ mice was verified by immunoblotting and qPCR (Figure S1). In the wild-type mice, qRT-PCR analysis showed increased expression of legumain mRNA in the liver and spleen from the 25(OH)D_3_-treated compared to control mice (Figure 2A). In addition, immunoblot analysis showed a tendency towards increased level of 36 kDa mature legumain in the kidneys, liver, and spleen of 25(OH)D_3_-treated mice, although not statistically significant (Figure 2B,C). No prolegumain (56 kDa) was observed in these organs. Furthermore, an increased level of legumain proteolytic activity was detected in the kidneys of the 25(OH)D_3_-treated mice compared to control mice (Figure 2D). Importantly, ELISA measurement of legumain in the plasma revealed increased circulating legumain levels in mice treated with 25(OH)D_3_ versus control (Figure 2E). Plasma levels of VD_3_ metabolites in 25(OH)D_3_ and vehicle-treated mice were also measured and showed a positive correlation between the level of 1,25(OH)_2_D_3_ and circulating legumain (Figure 2F).

### 3.3. Legumain Cleaves VDBP In Vitro and In Vivo

**Figure 3:**
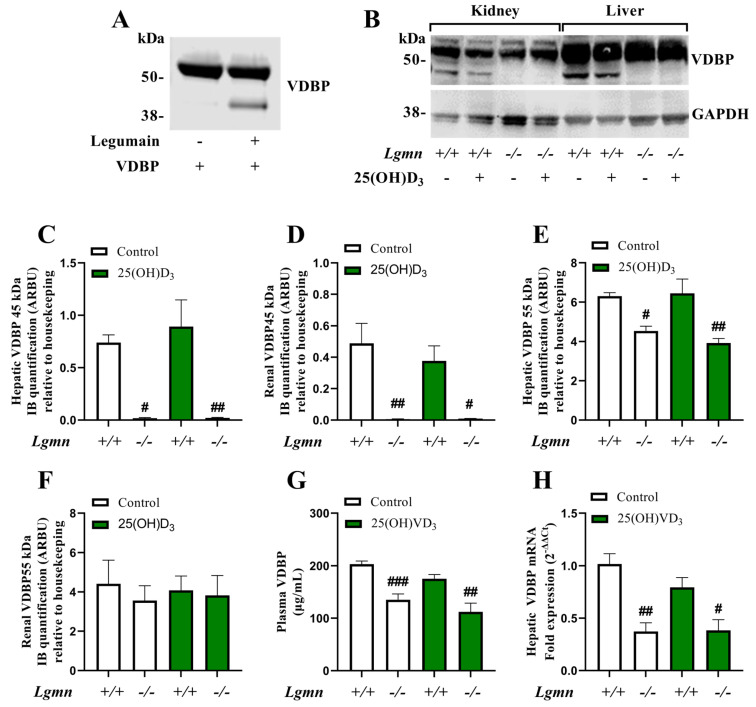
**Legumain is required for VDBP processing and regulation**. (**A**) Purified VDBP from human plasma (1.9 μM) was incubated in legumain assay buffer (pH 5.8) at 37 °C with or without purified active bovine legumain (2 μM) for 5 h before gel electrophoresis and immunoblotting of VDBP (n = 1). (**B**–**H**) Wild-type (*Lgmn*^+/+^) and legumain-deficient (*Lgmn*^−/−^) mice were treated with 50 µg/kg 25(OH)D_3_ (n = 6–7) or an equal volume vehicle (n = 7, control) subcutaneously every two to three days (four times in total). Tissues were harvested 24 h after the final injection (day 8). (**B**) One representative immunoblot of VDBP and GAPDH (housekeeping) in kidney and liver (n = 4). (**C**–**F**) Quantification of VDBP immunoband (IB) intensity as arbitrary units (ARBU) relative to GAPDH in immunoblots represented in (**B**) (n = 4). (**C**) Hepatic VDBP 45 kDa immunoband. (**D**) Renal VDBP 45 kDa immunoband. (**E**) Hepatic VDBP 55 kDa immunoband. (**F**) Renal VDBP 55 kDa immunoband. (**G**) Plasma VDBP concentration (μg/mL) was measured by ELISA (n = 6–7). (**H**) Hepatic VDBP mRNA expression relative to the geometric mean of CT values of four housekeeping controls (2^−ΔΔCT^, n = 5). (**C**–**H**) Data represent mean ± SEM. Two-way ANOVA. # *p* < 0.05, ## *p* < 0.01, ### *p* < 0.001 vs. different genotype, same treatment. Numbers (n) represent individual biological replicates.

VDBP has previously been reported as a legumain substrate [22]; thus, we aimed to investigate the possible role of legumain in the regulation of VD_3_ metabolism. First, we examined VDBP processing by legumain using incubation of purified VDBP from human plasma with or without purified active bovine legumain, followed by immunoblot analysis. Cleavage of full-length VDBP (55 kDa) by active legumain generated a VDBP cleavage product of approximately 45 kDa, which was not observed in the absence of legumain (Figure 3A). In addition, purified VDBP was incubated with or without lysate from legumain over-expressing HEK293 (M38L) cells [26], and a similar cleavage product (~45 kDa) was detected (Figure S2).

To further investigate the role of legumain in VDBP processing in vivo, the abovementioned wild-type (*Lgmn*^+/+^) and legumain-deficient (*Lgmn*^−/−^) mice were treated (sc) with 25(OH)D_3_ or vehicle for 8 days. Immunoblot analysis of VDBP in homogenates from the liver and kidney of *Lgmn*^−/−^ mice did not show the generation of the 45 kDa VDBP cleavage product compared to the wild-type control (*Lgmn*^+/+^) mice (Figure 3B–D). Interestingly, significantly decreased expression of full-length VDBP was detected in the liver from *Lgmn*^−/−^ compared to control mice, as observed by immunoblotting (Figure 3B,E), whereas legumain deficiency did not alter the levels of full-length VDBP in the kidneys (Figure 3B,F). No effect of 25(OH)D_3_ treatment on VDBP levels or its processing was observed in kidneys or liver from either *Lgmn*^+/+^ or *Lgmn*^−/−^ mice (Figure 3B–F). The level of VDBP in plasma was analyzed using ELISA and showed decreased circulating VDBP levels in *Lgmn*^−/−^ compared to *Lgmn*^+/+^ mice (Figure 3G). In addition, qRT-PCR analysis revealed a significantly decreased level of VDBP mRNA expression in the liver from *Lgmn*^−/−^ mice (Figure 3H). We also observed a tendency towards decreased levels of VDBP in plasma and VDBP mRNA expression in the liver from 25(OH)D_3_-treated wild-type mice (Figure 3G and Figure 3H, respectively).

### 3.4. Legumain Deficiency Alters Vitamin D Metabolism In Vivo

**Figure 4:**
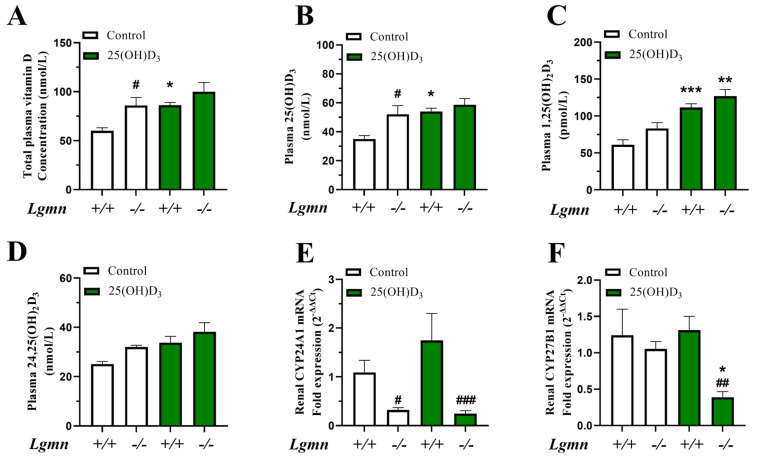
**Legumain deficiency alters plasma levels of vitamin D metabolites and induces changes in renal expression of vitamin D-metabolizing enzymes.** Wild-type (*Lgmn*^+/+^) and legumain-deficient (*Lgmn*^−/−^) mice were treated with 50 µg/kg 25(OH)D_3_ (n = 6–7) or an equal volume vehicle (n = 7, control) subcutaneously every two to three days (four times in total). Tissues were harvested 24 h after the final injection (day 8). (**A**–**D**) Vitamin D_3_ metabolites in plasma were analyzed by LC-MS/MS. (**A**) Total plasma concentration of vitamin D_3_ metabolites (25(OH)D_3_, 1,25(OH)_2_D_3_, and 24,25(OH)_2_D_3_) (nmol/L, n = 4–5). (**B**) Plasma 25(OH)D_3_ concentration (nmol/L, n = 5). (**C**) Plasma 1,25(OH)_2_D_3_ concentration (pmol/L, n = 4–5). (**D**) Plasma 24,25(OH)_2_D_3_ concentration (nmol/L, n = 5). (**E**,**F**) Renal CYP24A1 (**E**) and CYP27B1 (**F**) mRNA expressions relative to the geometric mean of CT values of four housekeeping controls (2^−ΔΔCT^; n = 5). Data represent mean ± SEM. (**A**–**D**). Two-way ANOVA. (**E**,**F**) Two-way ANOVA on ΔCT values. * *p* < 0.05, ** *p* < 0.01, *** *p* < 0.001 vs. same genotype, different treatment. # *p* < 0.05, ## *p* < 0.01, ### *p* < 0.001 vs. different genotype, same treatment. Numbers (n) represent individual biological replicates.

To examine VD_3_ metabolism in *Lgmn*^+/+^ versus *Lgmn*^−/−^ mice, LC-MS/MS technology was employed to determine the circulating levels of VD_3_ metabolites. Interestingly, increased basal plasma levels of total VD_3_ and 25(OH)D_3_, as well as a tendency towards increased 1,25(OH)_2_D_3_ and 24,25(OH)_2_D_3_ levels, were found in *Lgmn*^−/−^ compared to *Lgmn*^+/+^ control mice (Figure 4A–D). As expected, 25(OH)D_3_ treatment increased the plasma levels of all VD_3_ metabolites in *Lgmn*^+/+^ mice. In addition, 25(OH)D_3_ treatment significantly increased the plasma level of 1,25(OH)_2_D_3_ in *Lgmn*^−/−^ mice (Figure 4C).

To further investigate the effect of legumain deficiency on VD_3_ metabolism, renal mRNA expressions of the two key metabolic enzymes CYP24A1 (24-hydroxylase) and CYP27B1 (1α-hydroxylase) were analyzed. Significantly decreased CYP24A1 mRNA was detected in kidneys obtained from *Lgmn*^−/−^ compared to *Lgmn*^+/+^ mice (Figure 4E). However, hepatic CYP24A1 mRNA expression increased significantly in *Lgmn*^−*/*−^ mice in response to 25(OH)D_3_ treatment, an effect that was not seen in *Lgmn*^+/+^ mice (Figure S3). 25(OH)D_3_ treatment did not have any effect on renal expression of CYP24A1 mRNA (Figure 4E). In addition, no difference in renal expression of CYP27B1 mRNA was observed in either *Lgmn*^−*/*−^ or *Lgmn*^+/+^ mice. However, after 25(OH)D_3_ administration, a significantly decreased renal level of CYP27B1 mRNA was detected in *Lgmn*^−*/*−^ compared to *Lgmn*^+/+^ mice (Figure 4F).

## 4. Discussion

In the present study, VD_3_ was identified as an inducer of legumain expression and proteolytic activity in pre-osteoblasts and mouse tissues. In addition, the cleavage of VDBP by legumain was, for the first time, demonstrated in vivo. Interestingly, legumain deficiency resulted in transcriptional downregulation of hepatic VDBP synthesis, resulting in reduced levels of circulating VDBP. Furthermore, legumain deficiency also altered VD_3_ metabolism due to changes in the renal expression of key metabolic enzymes (CYP27B1 and CYP24A1), resulting in altered basal levels of VD_3_ metabolites, as well as in response to 25(OH)D_3_ treatment.

Initially, in silico studies indicated the presence of vitamin D-responsive elements (VDRE) in the promoter region of the legumain encoding gene (*LGMN*). Therefore, we hypothesized that VD_3_ could be a regulator of legumain expression. Our cell-based studies using osteogenic cultures of human BMSC showed increased mRNA expression, proteolytic activity, and secretion of legumain by pre-osteoblasts in the presence of 1,25(OH)_2_D_3_. Although the promoter of the *LGMN* gene contains VDRE, it is most likely that the enhancing effect of VD_3_ on legumain expression is mediated through an indirect mechanism, as a direct transcriptional regulation of legumain expression by VD_3_ would likely result in a more pronounced effect.

We also observed increased legumain activity in the presence of the VD_3_ metabolite 25(OH)D_3_. This was likely due to the conversion of 25(OH)D_3_ to 1,25(OH)_2_D_3_ by the pre-osteoblasts, as CYP27B1 is expressed and functional in these cells [33,34,35]. In addition, administration of 25(OH)D_3_ to wild-type mice increased the expression and activity of legumain in various tissues and, importantly, increased the circulating levels of legumain in the plasma. These data provide strong evidence that VD_3_ is an upstream regulator of legumain expression. It has previously been shown that there is minimal overlap in genes regulated by VD_3_ between different cell types or species [36]. Interestingly, we observed that legumain was regulated in a similar manner in human pre-osteoblastic cells and mice. However, whether the functional and physiological consequences of VD_3_-induced production of legumain are conserved in mice and humans is currently not known.

Identification of VD_3_ as an upstream regulator of legumain expression provides new insights into the role of VD_3_ in modulating cellular processes beyond its well-known roles in, i.e., regulation of calcium homeostasis. The ability of VD_3_ to regulate legumain expression suggests a possible involvement of VD_3_ in legumain-mediated physiological and pathological processes. In this regard, and since legumain has an inhibitory role in osteoblast maturation [4], it is possible that legumain plays a role in the inhibition of osteoblast differentiation and reduction of bone mass associated with a high dose of VD_3_ administration [37,38,39].

The present study provides evidence that VDBP is processed by legumain both in vitro and in vivo, corroborating a previous study presenting VDBP as a legumain substrate [22]. We observed no VDBP processing in mouse kidneys or liver upon legumain deficiency, which could possibly lead to an increased level of VDBP in the circulation. However, interestingly, significantly lower plasma levels of VDBP were observed in *Lgmn*^−/−^ compared to *Lgmn*^+/+^ mice. Renal dysfunction manifested as decreased glomerular filtration rate, increased plasma creatinine, and fibrosis, and premature senescence has been demonstrated in legumain-deficient mice [40,41]. Whether the decrease in circulating VDBP levels in *Lgmn*^−/−^ mice is caused or exacerbated by proteinuria is not known. However, the present data show a significant decrease in mRNA and protein expressions of VDBP in the liver upon legumain deficiency, which suggests a negative regulatory feedback loop that ensures decreased hepatic production of VDBP to counteract the systemic lack of VDBP processing by legumain. In addition, the observed increase in total VD_3_ metabolite concentration in conjunction with decreased VDBP levels upon legumain deficiency indicates that proteinuria is not the cause of the reduced plasma VDBP level as the absolute majority of VD_3_ metabolites are bound to VDBP and would be excreted along with the carrier protein [42,43]. In a normal state, the plasma VDBP level is in a substantial surplus with regard to the VD_3_ metabolite levels, and the binding capacity of VDBP far exceeds the level of available VD_3_ metabolites [44,45,46]. In addition, VD_3_ metabolites are also bound to albumin, although to a lesser extent. Therefore, the observed increase in total VD_3_ metabolite concentration seen in *Lgmn*^−/−^ mice is not a contradiction to the decrease in plasma VDBP.

The mice used for in vivo experiments were kept on a regular diet (chow) with sufficient amounts of dietary VD_3_. Therefore, in order to provoke detectable changes in the levels of circulating VD_3_ metabolites, high doses of parenteral 25(OH)D_3_ were administered. However, the total exposure was within the range of what has previously been used in comparable experiments [47,48], and the detected levels of VD_3_ metabolites were well below what has been considered toxic [48]. Results in the present study showed increased plasma levels of total VD_3_ and 25(OH)D_3_ in *Lgmn*^−/−^ compared to *Lgmn*^+/+^ mice, which could be explained by reduced tissue distribution of VD_3_ or reduced clearance due to decreased levels of VDBP upon legumain deficiency. In addition, our data indicated a tendency towards increased plasma levels of 1,25(OH)_2_D_3_ in *Lgmn*^−/−^ mice, which could reflect increased total VD_3_ and 25(OH)D_3_ plasma levels upon legumain deficiency. However, the lack of major changes in the plasma levels of 1,25(OH)_2_D_3_ upon legumain deficiency indicates the presence of legumain-independent mechanisms that could play a role in the release of VD_3_ from VDBP in the kidneys, which is required for hydroxylation to the active 1,25(OH)_2_D_3_.

We observed a significantly decreased level of CYP24A1 mRNA expression in kidneys from *Lgmn*^−/−^ mice. However, it is intriguing that the plasma level of 24,25(OH)_2_D_3_ did not decrease in these mice. As 24,25(OH)_2_D_3_ is generated by CYP24A1-mediated hydroxylation of 25(OH)D_3_, the lack of change in the plasma levels of 24,25(OH)_2_D_3_ in *Lgmn*^−/−^ mice could be due to the increased plasma levels of 25(OH)D_3_ upon legumain deficiency, together with CYP24A1-mediated hydroxylation of 25(OH)D_3_ in extra-renal vitamin D-targeted tissues. This notion is supported by studies indicating that extra-renal CYP enzymes are involved in the regulation of VD_3_ metabolism [49,50,51] and the increase in hepatic CYP24A1 mRNA expression in 25(OH)D_3_-treated *Lgmn*^−/−^ mice. It has recently been shown that extra-renal CYP24A1 ameliorates severe hypercalcemia in mice with kidney-specific CYP24A1 ablation [52].

CYP27B1 is the key enzyme involved in the hydroxylation of 25(OH)D_3_ and the production of active 1,25(OH)_2_D_3_. Expression of CYP27B1 mRNA in kidneys of *Lgmn*^−/−^ mice was significantly decreased upon 25(OH)D_3_ administration. Taking into account the increased plasma levels of total VD_3_ and 25(OH)D_3_ in *Lgmn*^−/−^ mice, together with significantly decreased expression of the VD_3_ catabolizing enzyme CYP24A1 upon legumain deficiency, decreased renal expression of CYP27B1 mRNA in *Lgmn*^−/−^ mice could be a feedback mechanism to avoid high levels of 1,25(OH)_2_D_3_ production and its associated side effects such as hypercalcemia [53]. This is in line with a previous study indicating decreased renal expression of CYP27B1 in mice that are unable to catabolize VD_3_ due to CYP24A1 deficiency [53].

## 5. Conclusions

**Figure 5:**
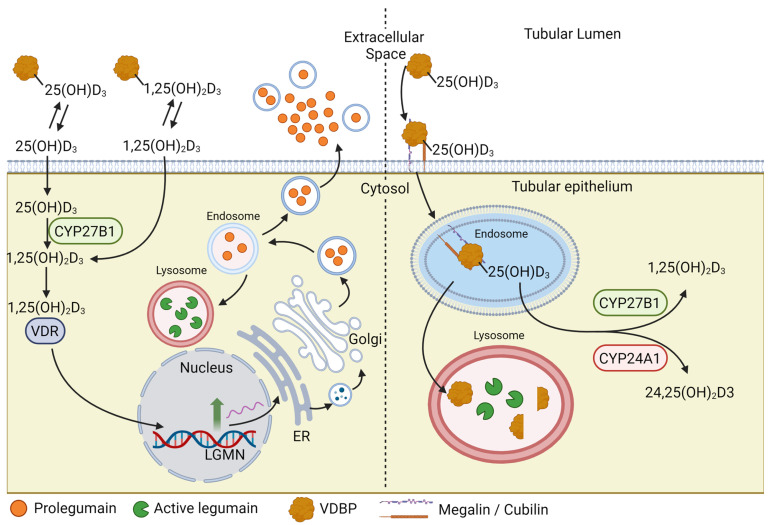
**Graphical representation of the suggested interplay between vitamin D and legumain. Left panel**: Vitamin D (VD_3_) promotes legumain expression and activity through transcriptional upregulation of the legumain gene (LGMN). The free fraction of circulating VD_3_ metabolites diffuse through plasma membranes. 25-hydroxyvitamin D (25(OH)D_3_) is hydroxylated by 1α-hydroxylase (CYP27B1), forming the active metabolite 1α,25-dihydroxyvitamin D (1,25(OH)_2_D_3_). 1,25(OH)_2_D_3_ binds to the nuclear vitamin D receptor (VDR) and promotes transcription of legumain (LGMN). Synthesized prolegumain is either sorted and activated in the endolysosomal system or released to the extracellular environment. **Right panel**: In the proximal tubular epithelium, 25(OH)D_3_ bound to vitamin D binding protein (VDBP) is internalized from the tubular lumen through a megalin/cubilin-mediated process. The vitamin D metabolite is released, enabling subsequent hydroxylation by 1α-hydroxylase (CYP27B1) or 24-hydroxylase (CYP24A1), and VDBP is cleaved by legumain in the endolysosomal system. VDBP cleavage by legumain might be important in controlling the systemic level of vitamin D metabolites. Created with BioRender.com (accessed on 11 December 2023).

Overall, the present work revealed the role of VD_3_ as an upstream enhancer of legumain expression both in vitro and in vivo and that legumain plays a role in the regulation of VD_3_ metabolism. This suggests a potential feedback loop where legumain activity can modulate the bioavailability of VD_3_ and its metabolites and possibly its downstream physiological processes (Figure 5). These findings provide insight into the intricate relationship between VD_3_ and legumain and can possibly open new avenues for research and investigation of novel therapeutic opportunities in various diseases in which VD_3_ and legumain play crucial roles.

## Data Availability

Data are contained within the article.

## Supplementary Materials

The following supporting information can be downloaded at https://www.mdpi.com/article/10.3390/cells13010036/s1.
