# Supplementary material for: The Cysteine Protease Legumain Is Upregulated by Vitamin D and Is a Regulator of Vitamin D Metabolism in Mice
Source: Cells. 2023 Dec 22;13(1):36. doi: 10.3390/cells13010036 (PMC10778535; doi:10.3390/cells13010036)
Supplement: Supplementary file 1 [file cells-13-00036-s001.zip › cells-2760617-supplementary.pdf]

*Article*

# The Cysteine Protease Legumain Is Upregulated by Vitamin D and Is a Regulator of Vitamin D Metabolism in Mice

**Karl Martin Forbord [1,2], Meshail Okla [2,3], Ngoc Nguyen Lunde [1], Tatjana Bosnjak-Olsen [1], Guro Arnekleiv [1], Daniel Hesselson [4], Harald Thidemann Johansen [1], Jonathan C. Y. Tang [5,6], Moustapha Kassem [2,7], Rigmor Solberg [1,*,†] and Abbas Jafari [7,*,†]**

**Supplementary Materials:** The following supporting information can be downloaded at: www.mdpi.com/xxx/s1.

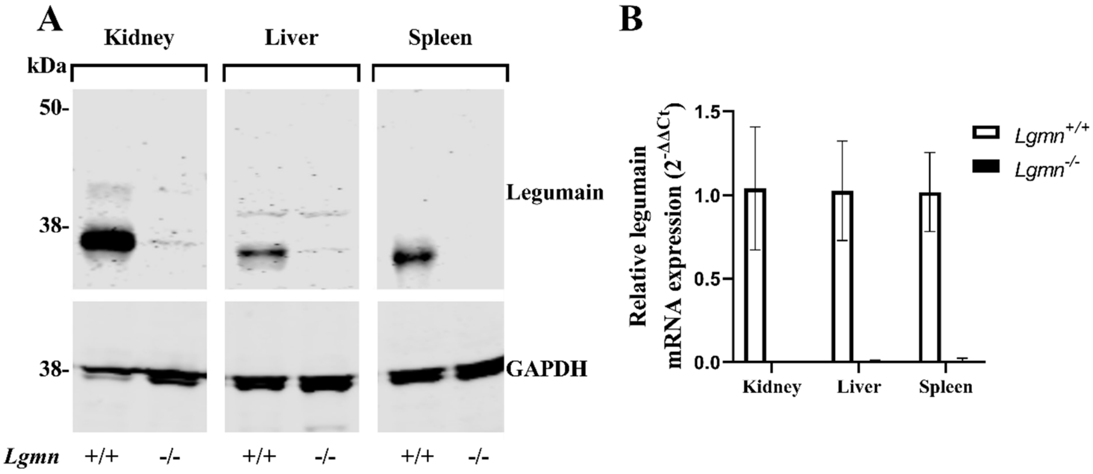


**Figure S1. Validation of legumain deficiency in Lgmn$^{-/-}$ mice.** Tissues from wild type (Lgmn$^{+/+}$) and legumain deficient (Lgmn$^{-/-}$) mice were harvested. **A.** One representative immunoblot of legumain and GAPDH (housekeeping control) in kidney, liver and spleen (n=3). **B.** Legumain mRNA expression relative to GAPDH (2-ΔΔCT; n=3). Data represent mean ± SEM. Numbers (n) represent individual biological replicates.

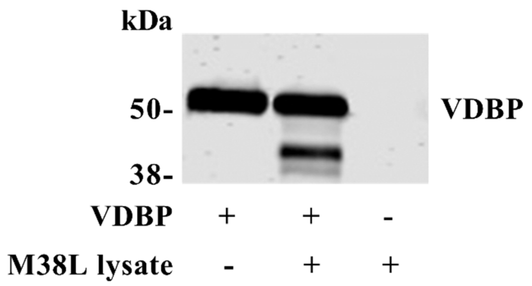


**Figure S2. VDBP is cleaved after incubation with lysate from legumain over-expressing HEK293 cells.** Purified VDBP (55 kDa) from human plasma (1.9 µM) was incubated in legumain assay buffer (pH 5.8) at 37 °C with or without lysate from legumain over-expressing HEK293 (M38L) cells for 5 hours (n=1).

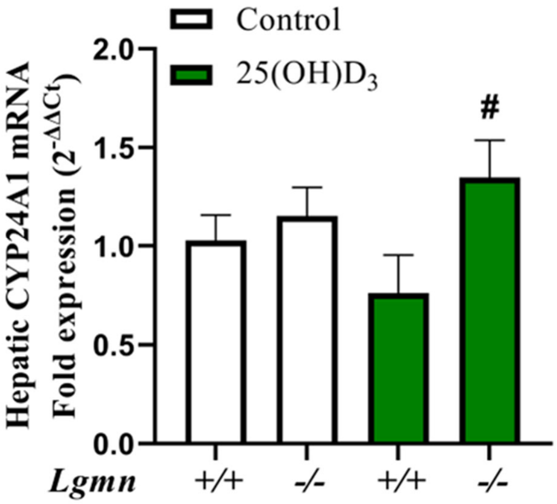


**Figure S3. Hepatic CYP24A1 mRNA expression is increased in *Lgmn*$^{-/-}$ mice treated with 25(OH)D$_3$.** Wild type (*Lgmn*$^{+/+}$) and legumain deficient (*Lgmn*$^{-/-}$) mice were treated subcutaneously with 50 μg/kg 25(OH)D3 (n=6-7) or an equal volume vehicle (n=7, control) every two to three days (four times in total) for seven days. Tissues were harvested 24 hours after the final injection. Hepatic CYP24A1 mRNA expression relative to the geometric mean of CT values of four housekeeping controls ($2^{-\Delta\Delta CT}$, n=5). Two-way ANOVA. #$p<0.05$ vs different genotype, same treatment. Numbers (n) represent individual biological replicates.

**Table S1.** Primer sequences used for qPCR.

| | | |
|---|---|---|
| **Human primer sequences** | | |
| GAPDH | Forward | GTCTCCTCTGACTTCAACAGCG |
| | Reverse | ACCACCCTGTTGCTGTAGCCAA |
| LGMN | Forward | GCAGGTTCAAATGGCTGGTAT |
| | Reverse | GGAGTGGGATTGTCTTCAGAGT |
| **Mouse primer sequences** | | |
| *18s* | Forward | GGGTCGGGAGTGGGTAATTT |
| | Reverse | GGGAGCCTGAGAAACGGC |
| *β-actin* | Forward | GATATCGCTGCGCTGGTCGTC |
| | Reverse | ACGCAGCTCATTGTAGAAGGTGTGG |
| *Cyp24a1* | Forward | CTGCCCCATTGACAAAAGGC |
| | Reverse | CTCACCGTCGGTCATCAGC |
| *Cyp27b1* | Forward | TCCTGGCTGAACTCTTCTGC |
| | Reverse | CCAGACCATATTGGCCCGTA |
| *Gapdh* | Forward | CATCACTGCCACCCAGAAGACTG |
| | Reverse | ATGCCAGTGAGCTTCCCGTTCAG |
| *Lgmn* | Forward | TGGACGATCCCGAGGATGG |
| | Reverse | GTGGATGATCTGGTAGGCGT |
| *Rplp0* | Forward | GCTTCGTGTTCACCAAGGAGGA |
| | Reverse | GTCCTAGACCAGTGTTCTGAGC |
| *Vdbp* | Forward | ACACCCAACACCTCTCCGGCA |
| | Reverse | CCAAGCTAGTGCACGGGCCC |
